# NAA and BOLD dynamics after single short stimulus in motor cortex of schizophrenia patients

**DOI:** 10.1192/j.eurpsy.2023.657

**Published:** 2023-07-19

**Authors:** M. Ublinskiy, A. Manzhurtsev, T. Akhadov, I. Lebedeva

**Affiliations:** ^1^Radiology, Clinical And Research Institute Of Emergency Pediatric Surgery And Trauma; ^2^Psychiatry- National Mental Health Research Centre, Moscow, Russian Federation

## Abstract

**Introduction:**

Endogenous psychoses, e.g. schizophrenia, are a pressing problem of modern medicine and biology. Among various neurobiological models of schizophrenia,much attention is paid to disturbances in the brain neural activity and metabolism.

**Objectives:**

The aim of this study was to analyze dynamics of motor cortex metabolites in the norm and in early stage of schizophrenia in period of BOLD response to event related single stimulus using MRI methods (fMRI and NMR).

**Methods:**

Study was performed on clinical Phillips Achieva 3.0 T MRI scanner. Volume of interest in motor cortex was localized on the base of fMRI study (EPI FFE, TR = 3000 ms, TE = 30 ms) as the zone of activation (Fig 1) caused by bottom push with the forefinger in response to single auditory stimuli transmitted with the 18 s periodicity. The BOLD signal was measured each 3 sec. 1Н МR spectra (PRESS, TE = 30 ms TR = 3000 ms) were run; FID signals for time points t = 0, 3, 6, 9, 12, 15, 18 s after stimulus were summarized (Fig 2). Thus, the synchronization of BOLD and metabolic responses to single stimulus was achieved. The same method was applied for spectra accumulation in resting state. For FID processing custom made software was used (with apodization filtering (LB = 20, GB = -5), FT and manual phase correction).

NAA, Cho, Cr signal intensities for each time point were normalized to their values at t = 0 and to the volume of activated cells containing in the voxel (segmented manually). Intergroup difference and time points differences were estimated using Mann-Whitney criterion with the level of significance p<0.05.

**Results:**

The BOLD signal in both groups demonstrated maximum at the 6th s after target stimulus, however its value was reliably lover in schizophrenia in comparison with the control group.

The only [NAA] in normal motor cortex was changed after the stimulation (Fig D). In schizophrenia [NAA], [Cr] and [Cho] were constant. The stable values of [NAA], [Cr] and [Cho] were observed in dynamics in resting state as well. [NAA] in normal cortex statistically significantly decreased at the 12th s after stimulus presentation and returned to initial value at the 15th s (Fig 3). Thus [NAA] minimum delayed relative to maximum of BOLD by 6 s.

**Image:**

**Figure:**
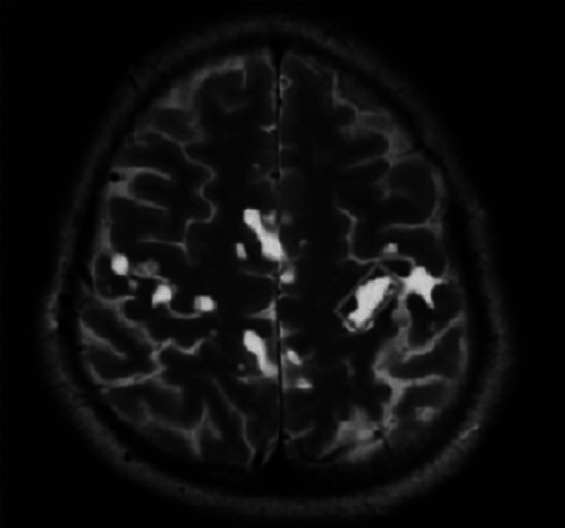

**Image 2:**

**Figure:**
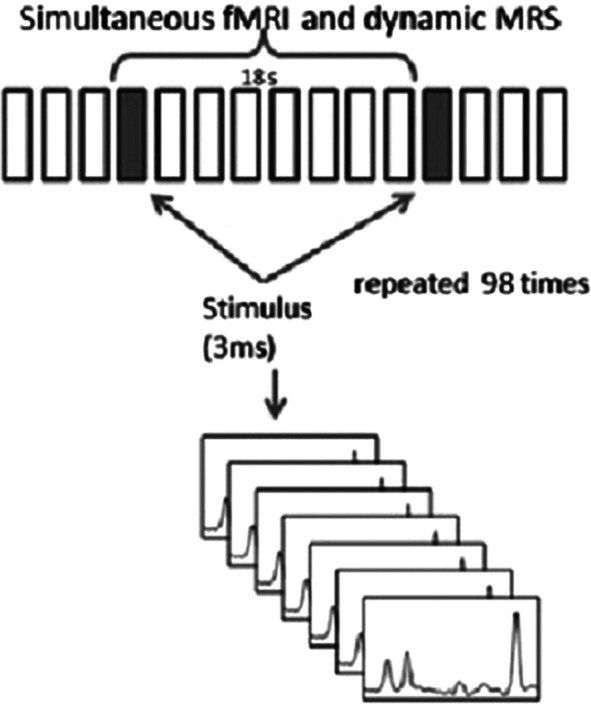

**Image 3:**

**Figure:**
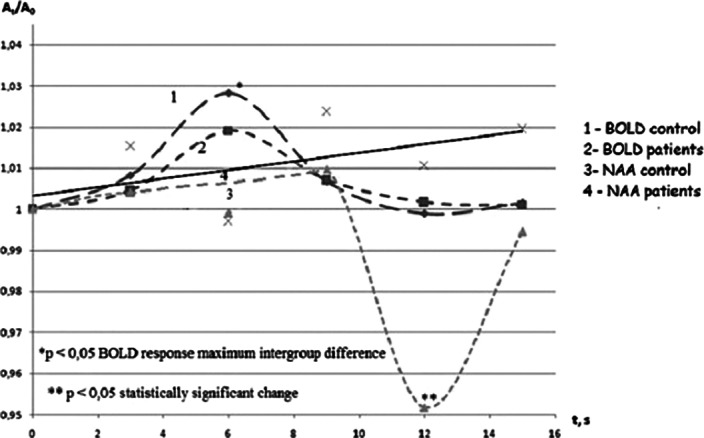

**Conclusions:**

The reversible decrease of NAA observed for the norm in the study could provide a short-term activation of neuronal Krebs cycle through a synthesis of Ac CoA using acetate obtained in ASPA reaction. Different behavior of [NAA] in the norm and schizophrenia might be related with a difference in location (or activity) of ASPA. Decreased expression of glutamate transporters in schizophrenia could also reduce consumption of NAA as a source of acetate in synthesis of Ac CoA which is used for restoration of ATP.

**Disclosure of Interest:**

None Declared

